# Rhabdomyosarcome embryonnaire du cuir chevelu: à propos d’un cas

**DOI:** 10.11604/pamj.2018.29.23.14281

**Published:** 2018-01-11

**Authors:** Othmane Alaoui

**Affiliations:** 1 Service de Chirurgie Pédiatrique Viscérale, CHU Hassan II, Université Sidi Mohamed Ben Abdallah, Fès, Maroc

**Keywords:** Rhabdomyosarcome, cuir chevelu, enfant, Rhabdomyosarcoma, scalp, child

## Abstract

We report the case of a newborn male admitted on the first day of his life with temporo-occipital swelling detected at birth. Clinical examination showed conscious, hemodynamically and respiratory stable, afebrile, vigorous and responsive newborn; craniofacial examination objectified soft, mobile, temporo-occipital mass covered by the scalp measuring 10 cm along its longer left paramedian axis (A). Craniocerebral CT scan showed large heterogeneous left subgaleal parietal-temporal-occipital mass measuring 40/80 mm, suggesting a tumor of soft tissue components of the scalp (B). The newborn underwent surgery which revealed bilobed tumor of the left temporo occipital soft tissue components, without extension to the cranial or the endocranial vault (C). Complete resection of the two masses was performed (D). Anatomo-pathologic study showed histological and immunohistochemical features of embryonal rhabdomyosarcoma. Staging evaluation was unremarkable. No chemotherapy or radiotherapy was proposed to the patient. At 1-year follow-up the child was asymptomatic and ultrasound examination of the surgical site was unremarkable.

## Image en médecine

Nous rapportons le cas d’un nouveau né de sexe masculin, admis à J1 de vie, pour une tuméfaction temporo occipitale découverte à la naissance. L’examen clinique a trouvé un nouveau né tonique et réactif, conscient, stable sur le plan hémodynamique et respiratoire, apyrétique; avec à l’examen cranio facial une masse temporo-occipitale de 10cm de grand axe paramédiane gauche de consistance molle, mobile, recouverte par du cuir chevelu (A). La TDM cranio cérébral a montré une volumineuse formation sous galéale temporo parieto occipitale gauche hétérogène mesurant 40/80mm faisant évoquer une pathologie tumorale des parties molles du scalp (B). Le nouveau né a été opéré avec à l’exploration une tumeur au dépend des parties molles temporo occipitale gauche, bilobée, sans extension à la voute crânienne ni en endocrânien (C). Une résection tumorale complète en deux masses a été réalisée (D). L’étude anatomopathologique a objectivé un aspect histologique et immunohistochimique d’un rhabdomyosarcome embryonnaire. Le bilan d’extension était sans particularités. Aucune chimiothérapie ni radiothérapie n’a été proposé au malade Le recul est actuellement de 1 an; l’enfant est asymptomatique; l’échographie du site opératoire est sans particularités.

**Figure 1:**
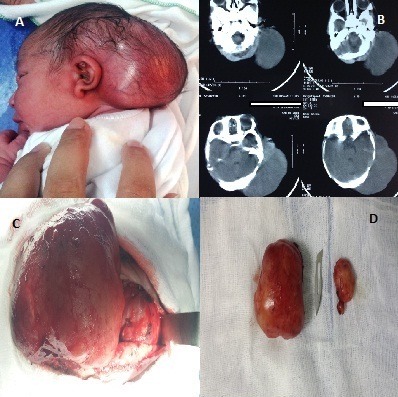
A) image clinique de la tumeur du cuir chevelu; B) image TDM de la tumeur du cuir chevelu; C) image peropératoire de la tumeur; D) pièce opératoire de la tumeur

